# Spectacles and How to Choose Them

**Published:** 1881-06

**Authors:** 


SPECTACLES AND HOW TO CHOOSE THEM. An elementary monograph, by C. A. Vilas, M.A., M.D. Professor of Diseases of the Eye and Ear in the Hahnemann Medical College and hospital,

Chicago, Illinois, etc., etc.,. Pp. 160. 12mo. Cloth. Price, $1. Duncan Brothers. 1881.

This work—intended to take its place beside those issued so largely of late from the medical press for non-professional, as well as professional readers—while offering nothing new, contains much that is of value. The general practitioner needs but go over its one hundred and sixty pages to find how little he knows of a subject that is of so much importance to both physician and patient. The different kinds of spectacle and eye-glass frames in use, with the lenses used for correcting the various forms of ametropia, are fully described: also the mode of procedure in such cases. A marked omission occurs in not naming the mydriatics that have come into use of late, as they have been found superior to atropine, which is mentioned, in being quicker in action, and the discomforts arising from their use are of much less duration. Attention to the various subjects of which it treats will make its possession of value to all physicians.

